# Coexisting intramedullary schwannoma with an ependymal cyst of the conus medullaris: A case report

**DOI:** 10.3892/ol.2014.2786

**Published:** 2014-12-10

**Authors:** TAO YANG, LIANG WU, CHENLONG YANG, XIAOFENG DENG, YULUN XU

**Affiliations:** Department of Neurosurgery, China National Clinical Research Center for Neurological Diseases, Beijing Tiantan Hospital, Capital Medical University, Beijing 100050, P.R. China

**Keywords:** conus medullaris, ependymal cyst, intramedullary, schwannoma

## Abstract

Synchronous spinal intramedullary ependymal cysts and intramedullary schwannomas are rare. To the best of our knowledge, the present study is the first report of a case of intramedullary schwannoma coexisting with an ependymal cyst. A 35-year-old male presented with lower back pain and weakness in the left leg. Magnetic resonance imaging identified an intramedullary cystic-solid lesion at the thoracolumbar junction of T11-L2; based on the clinical presentation and radiological features, a pre-operative diagnosis of ependymoma was formed. Subsequently, the patient underwent a T11-12 laminectomy via a posterior approach, with intraoperative monitoring of somatosensory and motor-evoked potentials, achieving a gross total resection of the tumor with a well-demarcated dissection plane. Post-operative histopathological examination demonstrated a schwannoma coexisting with the ependymal cyst, and the neurological status of the patient markedly improved compared with the pre-operatively observed neurological deficit.

## Introduction

Spinal schwannomas are usually intradural extramedullary tumors. As Schwann cells are not typically found in the parenchyma of the central nervous system (CNS), these tumors are rarely observed within the spinal cord (1). Intramedullary schwannomas account for 0.3% of intraspinal tumors and 1.1% of intraspinal schwannomas (2,3). Definitive preoperative differentiation from other intramedullary tumors based solely on imaging is impossible, unless there is a predominant extramedullary component or when the tumor is continuous with a thickened spinal nerve root (4,5). Since intramedullary schwannomas are slow growing and benign tumors, gross total resection (GTR) of the tumors is the primary treatment choice with good outcome (3). Ependymal cysts are ependymal-lined fluid collections within the CNS, the majority of which are generally located in the paraventricular white matter of the frontal and parietal lobes (6). Intramedullary ependymal cysts are particularly rare, with few pathologically determined cases to date (7). Although these cysts are completely independent from the central canal, only 28% of intramedullary ependymal cysts could be totally resected (7). Thus, complete decompression and cyst-subarachnoid shunt placement is the optimal treatment, and the outcome may be favorable (8). In the literature, only one case has been reported in association with a filar lipoma in an infant (9). The present study reports a case of intramedullary schwannoma coexisting with an ependymal cyst in an adult patient. Written informed consent for the publication of this study was obtained from the patient.

## Case report

On March 4, 2013, a 35-year-old male was admitted to Beijing Tiantan Hospital (Beijing, China) with a two-year history of lower back pain and weakness in the left leg. In addition, the patient had experienced sphincter dysfunction for two months. The patient had no history of spinal cord injury or previous back surgery. Neurological examination revealed the power in the left leg to be 3/5, as classified by the Medical Research Council grading system (10). Superficial sensation in the perineum and left leg was reduced.

**Figure 1:**
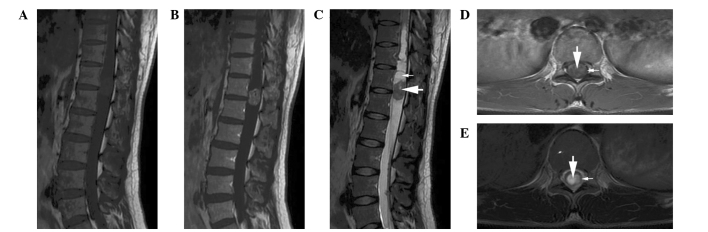
Pre-operative magnetic resonance imaging. (A) Sagittal T1-weighted image revealing an intramedullary cystic-solid lesion in the conus medullaris. (B) Solid lesion demonstrating inhomogeneous enhancement following gadolinium injection. (C) T2-weighted image revealing the solid mass (large arrow) accompanied by a cranial cystic lesion (small arrow). (D) Coronary contrast-enhanced T1-weighted image showing the solid mass (large arrow) located exclusively within the conus medullaris (small arrow). (E) Coronary T2-weighted image indicating the additional cranial cystic lesion (large arrow) present in the spinal cord (small arrow).

The patient initially underwent pre-operative magnetic resonance imaging (MRI; Fig. 1). Sagittal T1-weighted images demonstrated an intramedullary cystic-solid lesion in the conus medullaris; following gadolinium injection, the solid mass demonstrated inhomogeneous enhancement. Furthermore, T2-weighted images revealed that the solid mass was accompanied by a cranial cystic lesion. According to the pre-operative MRI, a diagnosis of ependymoma with cranial syringomyelia or cystic degeneration was determined.

The patient underwent a T11-12 laminectomy via the posterior approach with intraoperative monitoring of somatosensory and motor-evoked potentials. Intradural exploration revealed a bulging conus medullaris. Subsequently, classic median myelotomy was performed in the conus medullaris for intramedullary exploration. The solid mass was nodular in shape, with a poor blood supply. The mass was well demarcated from the spinal cord parenchyma and not attached by any nerve root, which facilitated its exposure and dissection. Following GTR of the solid mass, the cranial syringomyelia was examined. A cyst filled with clear fluid was identified; the cyst was in close proximity to the solid mass, however, it was separated by neural tissue. The cyst appeared yellow in color, was filled with clear fluid and did not adhere to the spinal cord, therefore, a GTR of the cyst was performed.

**Figure 2:**
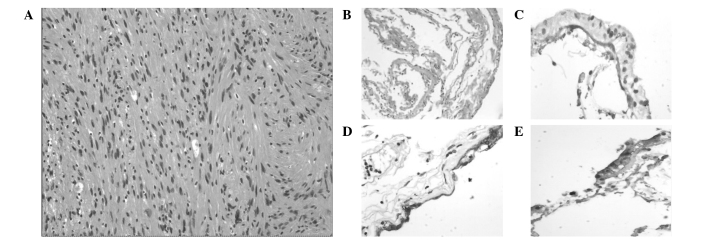
Photomicrographs of the solid mass illustrating (A) a benign spindle cell neoplasm with palisading of bland, vesicular nuclei consistent with a schwannoma (hematoxylin and eosin stain; original magnification, ×200); (B) the cyst wall, consisting of glial cells lined by a simple cuboidal to columnar epithelium (hematoxylin and eosin stain; original magnification, ×100); and positive staining for (C) cytokeratin, (D) glial fibrillary acidic protein and (E) S-100 protein (original magnification, ×200).

Pathological examination of the solid mass identified spindle-shaped cells with features of a schwannoma (Fig. 2A). Surgical specimens obtained from the cyst wall were examined and thin ependymal cells were identified to line the cavity (Fig. 2B). An immunohistochemical examination revealed that the cells lining the cyst wall were positive for cytokeratin, glial fibrillary acidic protein and S-100 protein expression (Fig. 2C–E).

**Figure 3:**
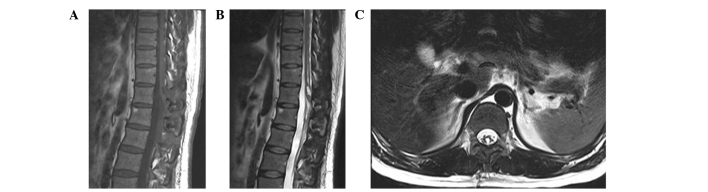
Contrast-enhanced (A) T1-weighted, (B) T2-weighted and (C) coronary T2-weighted magentic resonance images at nine months postsurgery showing no recurrence of the schwannoma or the cyst, and indicating the cavity left by the removal of the mass in the spinal cord.

In the immediate post-operative period, although the weakness in the left leg and the sphincter dysfunction did not improve, the lower back pain disappeared. The patient was discharged two weeks later; the weakness in the left leg had improved to grade 5/5 and sphincter dysfunction gradually improved after three months. No recurrence of the schwannoma or the cyst were observed on the follow-up MRI (Fig. 3). The present retrospective study was approved by the Institutional Review Board of Beijing Tiantan Hospital.

## Discussion

Schwannomas and ependymal cysts represent two distinct disease entities. To the best of our knowledge, intramedullary ependymal cysts have not previously been reported in association with schwannomas. Intramedullary schwannomas are predominantly located in the cervical region, followed by the thoracic region and the lumbar cord (11,12); the conus medullaris is rarely involved. According to the location in the cross-section of the spinal cord, intramedullary schwannomas can be divided into three types: Central, surfacing and exophytic (13). In the present case, the tumor was located exclusively within the spinal cord and, thus, was classified as a central-type intramedullary schwannoma. A central-type tumor with no association to the posterior nerve root is considered to be a congenital neoplasm, which consists of ectopic Schwann cells originating from the embryonic neural ridge during closure of the neural tube at the fourth week (13,14). Pain is always the initial symptom (12), and sensory and motor dysfunction appear in the late stages of the disease.

Intramedullary schwannomas have no specific imaging features, and are hypointense or isointense on T1-weighted images and generally hyperintense on T2-weighted images. Furthermore, heterogeneous enhancement and well-defined margins of the tumor on T1-weighted images are regarded as characteristic, but not specific for schwannomas (15,16). Therefore, unless a predominantly extramedullary component or nerve root thickening is observed, a definitive pre-operative diagnosis of intramedullary schwannoma is difficult to determine based solely on MRI data (4,5). However, only 50% of intramedullary schwannomas are directly connected with nerve roots (17). Hence, performing GTR without sacrificing the nerve roots is feasible in the majority of intramedullary schwannoma cases. Subtotal removal is advisable to avoid unacceptable surgical complications if the tumor exhibits dense adhesion to the neural tissue. For example, Hida *et al* (18) described two cases of adherent intramedullary schwannoma treated with two-stage surgery; the residual tumor was removed completely, decreasing the morbidity of the two patients.

Ependymal cysts originate from isolated ependymal cells of the neuroectoderm, which are cut off from the neural tube in the developing embryo (9). The nature of ependymal cysts may cause them to occur anywhere along the craniospinal axis (19), however, they are predominantly located in the conus medullaris (20,21). The etiology of the conus medullaris predominance remains unclear, although one explanation may be that the ependymal structure is more often present in the conus medullaris in the process of embryogenesis (22). Upon pathological examination of the cyst wall, a continuous lining with a single layer of epithelial cells and no a basement membrane is observed (23–25). Intramedullary ependymal cyst is a well-circumscribed, homogeneous lesion with a smooth margin, the contents of which are isointense with cerebrospinal fluid on T1- and T2-weighted images and no enhancement on contrast-enhanced T1-weighted images (8,23–25). In the present case, the ependymal cyst did not exhibit typical imaging features, instead demonstrating features similar to syringomyelia. We hypothesize that the intramedullary schwannoma altered the configuration of the ependymal cyst. Surgery is the optimal treatment strategy for symptomatic patients; a range of surgical treatments have been utilized for ependymal cysts, including GTR, biopsy, partial resection, marsupialization of the cyst and cyst-subarachnoid shunts (19–21,23–25).

In the present case, the cyst was totally resected with a discernible plane between the spinal cord and the cyst wall. However, this is not always feasible, as the majority of cysts appear densely adherent to the neural tissue. A review by Park *et al* (7) demonstrated that only 28% of intramedullary ependymal cysts can be totally resected. Therefore, adequate decompression and communication between the cyst and subarachnoid space may be necessary (8). Recurrence of ependymal cysts is rare and neurological function is usually improved by surgical decompression.

Definitive differentiation from other intramedullary tumors (such as ependymoma, astrocytoma and hemangioblastoma) based solely on MRI is not viable pre-operatively. In the present study, the solid mass did not demonstrate the significant enhancement observed in hemangioblastoma and it presented with cranial syringomyelia or cystic degeneration, therefore, a pre-operative diagnosis of ependymoma was considered. Certain studies have diagnosed cranial or caudal cystic degeneration as simply non-neoplastic peritumoral syringomyelia (26,27). Entering or draining the syringomyelia during the course of resection should be avoided, as it would collapse following tumor removal (28). In addition, it has been reported that syringomyelia resolution is not affected by whether the syringomyelia cavity is entered (29). However, the present surgery explored the syringomyelia and identified an independent cyst, which was subsequently demonstrated to be an ependymal cyst.

In conclusion, the present study reports a rare condition in which the association between the two different congenital neoplasms may indicate the presence of dysembryogenesis as the causative mechanism. Owing to the rarity of concurrent intramedullary schwannoma and ependymal cysts, as well as a lack of clinical and imaging characteristics, intramedullary schwannoma is easily misdiagnosed as ependymoma. Therefore, tumor excision with exploration of the syringomyelia adjacent to the tumor is the recommended surgical strategy.
